# Rates of bone fractures in a cohort of HIV-infected adults in the UK

**DOI:** 10.1186/1758-2652-13-S4-P100

**Published:** 2010-11-08

**Authors:** A Samarawickrama, R Malik, M Fisher, Y Gilleece, K Walker-Bone

**Affiliations:** 1 Brighton and Sussex University Hospitals, Department of HIV and Genitourinary Medicine, Brighton, UK; 2 Brighton and Sussex University Hospitals, Department of Rheumatology, Brighton, UK; 3 Brighton and Sussex Medical School, Department of Infection and Inflammation, Brighton, UK

## Purpose of study

With combination antiretroviral therapy (cART), HIV-associated mortality rates have declined substantially in the UK. However HIV-infected adults are living longer to develop co-morbidities, including low bone mineral density (BMD). This study investigates fractures in a UK cohort of HIV-infected adults.

## Methods

A cross-sectional survey of 1050 HIV-infected adults from routine HIV clinics was conducted. Subjects completed a questionnaire about demography, lifestyle factors, fracture history and management, exposure to glucocorticoids and other drugs influencing bone metabolism. HIV details (duration and route of infection, disease stage, cART exposure and parameters of viral control) were recorded.

## Results

There were 859 useable replies (response rate 82%) from 775 men and 84 women: mean age 43 years (range 19-77 years) and 87% Caucasian. Mean duration of HIV infection was 8 years (range 0-23 years). Co-infection with hepatitis B occurred in 16 subjects and hepatitis C in 11. Overall, 125 (15%) subjects reported 200 fractures: 119 (15%) men and 6 (7%) women. Common fracture sites were forearm (n=65), tibia/fibula (n=29), hand/foot (n=22) and digit (n=19). Hip fractures occurred in 6 subjects and 2 had clinical vertebral fractures. A bimodal distribution of fracture was seen: 114 (57%) fractures occurred in subjects <25 years (peak age 7-12 years) and 33 (17%) fractures in those >40 years, with 8 (24%) in subjects >50 years. Among subjects >40 years with fractures (30 men and 3 women; 32 Caucasian), common fracture sites were forearm (n=6, mean age 48 years) and tibia/fibula (n=4, mean age 49 years); there was 1 hip fracture (age 46 years). 15% of these subjects reported oral glucocorticoid exposure (vs. 9% without fractures (p=ns)) and 79% had ever smoked (vs. 72% without fractures (p=ns)).

## Conclusions

This is the first epidemiological study of fractures in HIV-infected adults in the UK. There were 2 peaks of fracture incidence: <25 years and 40-60 years. Forearm fractures were the commonest but fractures at other 'typical' osteoporotic sites (vertebral and hip) were seen. The second peak occurred at a younger age than those reported in HIV-negative subjects. This is consistent with data showing accelerated immune senescence in HIV. Although the clinical importance of low BMD is yet to be fully evaluated, if fracture rates are increased, routine assessment of low BMD and fracture risk may be warranted in this relatively young population.

